# Cochleogram-Based Speech Emotion Recognition with the Cascade of Asymmetric Resonators with Fast-Acting Compression Using Time-Distributed Convolutional Long Short-Term Memory and Support Vector Machines

**DOI:** 10.3390/biomimetics10030167

**Published:** 2025-03-10

**Authors:** Cevahir Parlak

**Affiliations:** Department of Computer Engineering, Faculty of Engineering, Fenerbahçe University, 34758 İstanbul, Türkiye; cevahir.parlak@fbu.edu.tr

**Keywords:** speech emotion recognition, cascade of asymmetric resonators, deep neural networks, support vector machines, speech filter banks

## Abstract

Feature extraction is a crucial stage in speech emotion recognition applications, and filter banks with their related statistical functions are widely used for this purpose. Although Mel filters and MFCCs achieve outstanding results, they do not perfectly model the structure of the human ear, as they use a simplified mechanism to simulate the functioning of human cochlear structures. The Mel filters system is not a perfect representation of human hearing, but merely an engineering shortcut to suppress the pitch and low-frequency components, which have little use in traditional speech recognition applications. However, speech emotion recognition classification is heavily related to pitch and low-frequency component features. The newly tailored CARFAC 24 model is a sophisticated system for analyzing human speech and is designed to best simulate the functionalities of the human cochlea. In this study, we use the CARFAC 24 system for speech emotion recognition and compare it with state-of-the-art systems using speaker-independent studies conducted with Time-Distributed Convolutional LSTM networks and Support Vector Machines, with the use of the ASED and the NEMO emotional speech dataset. The results demonstrate that CARFAC 24 is a valuable alternative to Mel and MFCC features in speech emotion recognition applications.

## 1. Introduction

Speech, an ordinary, everyday task for humans, is a complex and intricate phenomenon. As our most important communication channel, it provides the listener with information about the speaker’s gender identity, personal identity, fundamental frequency, and cues as to the speaker’s emotional state. Speech emotion recognition (SER) is a very active research field, especially as it pertains to human–robot interaction. SER applications will enable robots to interact with humans more naturally and empathetically, making them more appealing. Studies modeling human speech attempt to imitate the auditory system by dividing the human hearing frequency range into specific regions called filter banks. The development history of these filter banks can be partitioned into three stages, starting with rounded exponentials (roex family), followed by gammatone filters (gammatone, gammachirp, all-pole gammatone, and one-zero gammatone), and finally the filter cascade models including all-pole filter cascades, pole-zero filter cascades, and pole-zero filter cascades+ models. Filter banks possess numerous useful properties, including easily controllable bandwidths; functioning that can be described in simple terms; a stable low-frequency tail; an asymmetric shape; easily tunable and customizable peak gain variations; and realistic impulse responses, both dynamic and parameterized. In addition to this, their phase characteristics and impulse response timing are quite realistic; they can be easily implemented as digital filters, making them highly suitable for computerized auditory scene analysis; and they can be connected and modeled by traveling wave dynamics. Cochlear filter banks have employed different filter shapes throughout their circuitous history. In the early days of filter banks, resonances were investigated, whereas in later stages, Gaussian, rectangular, and trapezoidal models became more prevalent [1].

Speech filter banks have been extensively studied, with numerous models proposed to elucidate the characteristic of the human hearing system. Common examples include Mel filters [2], Lyon filters [3], PLP [4], gammatone filters [5,6], Seneff filters [7], and Meddis hair cell models [8]. More recent research has placed greater emphasis on human cochlea modeling, with notable works in this area including those by Bruce [9], Hohmann [10], King [11], Relaño [12], Jepsen [13], Verhulst [14], and Zilany [15]. Gammatone filters are among the most common filtering models used to imitate the human hearing system. The outputs of gammatone filters can be used to predict the frequency response of specific locations on the basilar membrane, which corresponds to the center frequency of the related gammatone filter. Gammatone filters distribute the center frequencies along the frequency spectrum according to the ERB (Equivalent Rectangular Bandwidth) [6] scale proportional to the bandwidths of each filter. The Meddis hair cell model [8] is another popular model for describing the transduction of mechanical waves into neural impulses. Cochlear hair cells contain a liquid transmitter which moves between hair cells and the auditory nerve through a diffusive membrane. The Meddis model describes the formation, movement, and loss of the transmitting substance between the hair cells and auditory nerve. The amplitude of mechanical stimulations caused by the entering sound waves creates proportional fluctuations in the diffusibility of the moving transmitter substance. The transmitting substance contained within the synaptic cleft of auditory nerve is partially lost and whereas the remainder is continuously returned to the hair cells. These fluctuations construct a linear relation between the excitation potential and the amount of transmitter involved. A high quantity of transmitter substance is associated with a high probability of a neural spike. Zylany’s model is an advanced model of the synapse between inner hair cells and the auditory nerve. It takes the constant waveform pressure as input and applies three parallel filters. The first and second filters provide an input to the inner hair cells; these cells then output a signal to drive the functioning of the inner hair cell and auditory nerve. The parameters of Zylany’s model are tuned according to the basilar membrane structure. Bruce’s model expands upon Zylany’s work, enhancing the synapse and spike production. The first part of the model is the presynaptic portion, which incorporates a power law and fractional Gaussian noise [16]. Power law delineates the behavior of auditory nerve fibers in response to constantly fluctuating stimuli; the fibers adapt regardless of the duration of the stimulus rather than exhibiting a fixed time constant. The first stage of Bruce’s model also includes fractional Gaussian noise, inferring that spiking probability is highly dependent on the spiking time history. The second part of the model relates to the synaptic portion and describes the replenishment at the four synaptic vesicles and neuro-transmitter release [17].

**Figure 1:**
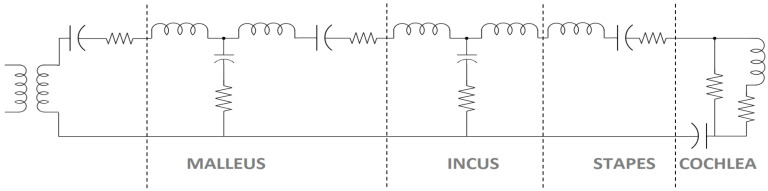
Electrical circuit diagram of artificial ear from outer ear through cochlea [24].

The human hearing system is perfectly compatible with the speech production system, possessing a unique structure that is tuned to optimally process vocal tract output. Sound waves entering the ear are amplified as they pass through the eardrum and middle ear, then directed to the inner ear for complex frequency analysis. During this analysis, the sound is separated into frequency ranges. Although cochlea structure modeling is ongoing, some aspects of cochlear mechanisms have yet to be fully elucidated. The inner ear, which is responsible for both hearing and balance, is located within the temporal bone and houses the vestibular system and cochlea. The vestibular system is used for balance, while the cochlea is responsible for hearing. These fluid-filled structures reside within a bony matrix. The hearing process begins with sound waves entering the ear canal and vibrating the eardrum. This vibration stimulates the middle ear ossicles: malleus, incus, and stapes. The oval window, where the stapes connects to the cochlea, is the site of mechanical force transfer. The cochlea is a snail-shaped, three-chamber structure, over 0.5 mm wide and 35 mm long, making 2.5 turns from the anterior part of the vestibulum around the modiolus, a bony pillar. In the cross-section, the cochlea has three channels: the Scala Vestibuli (SV), the scala tympani (ST), and the Scala Media (SM) between them. The base of the SV is the oval window, while the base of the ST is the round window. Perilymph fluid fills the SV and ST. The Corti, the sensory organ used for hearing, is located within the SM between the SV and ST. The SM contains the basilar membrane, tectorial membrane, hair cells, and endolymph fluid. Hair cells sense mechanical forces and convert them to neurological signals sent to the auditory cortex. Inner (IHCs) and outer hair cells (OHCs) are located between the tectorial and basilar membranes. Approximately 3500 IHCs are aligned in opposing rows, with more than 10,000 OHCs [18,19]. They can detect electrical signals up to approximately 20,000 Hz, the human hearing frequency threshold. While OHCs are more prevalent, it is IHCs that are crucial for hearing, with nearly 95% of afferent nerve fibers originating from them. IHCs function as receptors, establishing communication with the cranial nerve. Efferent signals are transmitted mostly to OHCs from the superior olivary complex. Stereocilia are located at the top of hair cells, connected by tiplinks, which enhance forces in molecular sensory areas. Sound travels along the SV and around the helicotrema to the ST, allowing perilymph fluid mixing. The signal continues its travel to the round window of the ST. The cochlea’s structure is not homogenous. When uncoiled, the base is stiffer than the tip; each part is sensitive to different frequencies. High frequencies stimulate the stiff base, while low frequencies are encoded by the soft apex. This creates a tonotopic map along the basilar membrane. The basilar membrane vibrates in response to sound; this knowledge is crucial for understanding the mechanical functioning of hearing. The force from the tectorial and basilar membrane movement causes hair cell oscillation, bending the stereocilia. The three chambers’ ionic environment is critical for signal transmission. The top of the hair cells is high in potassium, whereas the base is low in potassium. This imbalance opens up mechanosensitive channels, allowing potassium to enter and depolarize the cell. This depolarization opens calcium channels at the base, triggering neurotransmission to stimulate nerve fibers [18,19,20,21,22]. An electrical circuit equivalent of the human ear is depicted in Figure 1 [23,24,25]. Mathematical modeling of the human hearing system is studied in [26,27,28,29,30]. Zablotni et al. [31] studied the energy transfer within the inner ear between the round window and stapes. Electronic cochlea imitations, including vestibular prostheses [32] and artificial scala tympani [33], have also been investigated.

This paper is structured as follows: Section 1 introduces the concept of speech emotion recognition and its connection to the cochlear filter models. Section 2 reviews recent research on cochlear filters. Section 3 describes the role of the CARFAC 24 system in speech emotion recognition. Section 4 outlines the experimental setup of the study; the results of the experiments are presented in Section 5. Section 6 is reserved for limitations of CARFAC 24 system, Section 7 discusses the implications of the experimental findings, and finally, Section 8 summarizes the key conclusions of the manuscript and points to the directions of future works.

## 2. Related Studies

Xu et al. [34] investigated the performance of CARFAC (Cascade of Asymmetric Resonators with Fast-Acting Compression) with regard to sound localization, comparing the CARFAC 2011 and CAR 2011 methods with existing techniques and human performance. Their results demonstrated that CARFAC achieves comparable sound localization performance. They suggested that it may be applicable to audio source separation, reporting a non-quantized correlation error of 2.78 RMS at 0–45 degrees and 5.60 RMS at 45–90 degrees in the diagonal correlogram (500 Hz–8000 Hz). Xu et al. [35] also implemented a 70-channel CARFAC 2011 model on an FPGA, comparing it with other artificial cochlear models in terms of its frequency range, architecture, channel count, power consumption, and damping factor adjustment. While the CARFAC FPGA consumes more power than other models, it offers greater stability, scalability, ease of use, better compatibility with biological data, and an improved SNR.

Lyon [36] developed the CARFAC 2011 model, which better aligns with human-masked threshold data and can be readily modified for approximate-level-independent zero-crossing times. CARFAC effectively represents the cochlea’s nonlinear properties, leveraging its cascaded structure to mimic the traveling wave nature of sound in the cochlea. Its pole-zero filter uses multi-timescale-coupled automatic gain control and cascaded filter banks (unlike parallel banks in other models). These cascaded banks provide a better representation of sound wave propagation in the cochlea, with each layer modeling a specific cochlear region. The transfer function of each channel approximates the local sound wave using a pole-zero approach. The pole-zero filter cascade layers provide adaptive peak gain and pole damping, with the latter achieved by tuning signals from AGC networks that simulate outer hair cell feedback.

Zilany et al. [15] modified a phenomenological auditory peripheral model for cats, updating saturation rates in high-frequency bands and proposing a new analytical algorithm for calculating the variance and average discharge rate of synapse responses. These modifications integrated refractoriness, making the model more suitable for evaluating the higher-characteristic frequency (CF) neural encoding.

An alternative approach to traditional Fourier transform-based methods includes scalogram-based sound processing. Scarpiniti et al. [37] introduced Morlet wavelet-based scalograms to address the resolution limitations of Fourier transforms in Mel filter and MFCC [38] computations and assessed their approach on construction excavator, mixer, and compactor sounds using the AlexNet [39] model. When evaluated on 15-min recordings per class (five classes total), which were sliced into 30-millisecond chunks and split into 75% training and 25% test sets, their model achieved 98.9% precision and recall, outperforming previous studies.

All these models contribute to the development of better cochlear applications; however, CARFAC has a superior structure to the others, particularly compared to Mel filters. Mel filters are not treated as perfect models of the human cochlea, but merely as an engineering shortcut to facilitate the processing of speech signals. In contrast, CARFAC is directly inspired by the nature of human cochlea, and it incorporates all of the natural features of the human hearing system to accurately and robustly imitate and model the auditory system. It should be kept in mind that emotion recognition from speech is highly dependent on the low-frequency components. Most of the signals’ energy is confined to the low-frequency region of the human audible spectrum and different emotions will lead to different changes and fluctuations in the energy of each signal, facilitating the use of statistical functions. CARFAC is also a valuable tool for hearing emulation applications, in which a natural signal is important. Moreover, compared to the other gammatone and previous Lyon filters, CARFAC has a staged asymmetric resonator functioning. CARFAC’s temporal processing functioning is adaptive and faster than that of gammatone and Lyon systems. CARFAC also has an integrated internal amplitude compression system in which gammatone filters require external amplitude compression.

## 3. Proposed Approach

**Figure 2:**
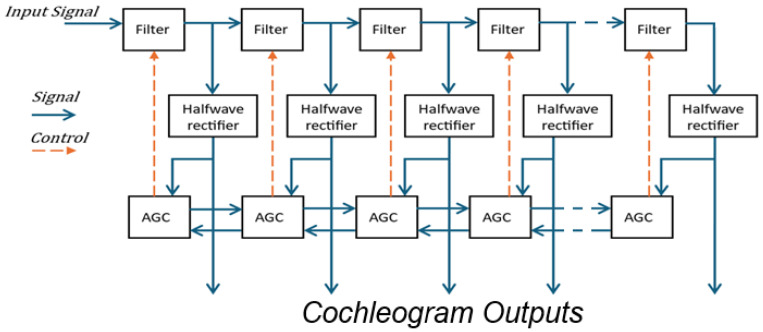
Block diagram of CARFAC system.

CARFAC, a highly advanced model, is based on the Lyon cochlear model created in 1982, and draws inspiration from the works of Schroeder [40] and Zweig [41]. Based on the Laplace domain pole-zero filter cascades (PZFCs), CARFAC has undergone numerous revisions, culminating in the 2011 and 2024 versions [1,36,42,43,44], which are designed to accurately replicate the neural and mechanical functions of the inner ear and align perfectly with the human cochlea. The general structure of the model is illustrated in Figure 2. CARFAC simulates the cochlea’s frequency analysis capabilities and converts signals into neurological codes. CARFAC comprises two fundamental components: the Cascade of Asymmetric Resonators (CARs) and Fast-Acting Compression (FAC). CARs models the resonant properties of the basilar membrane, distinguishing different frequency components of incoming sounds. Meanwhile, asymmetric resonators mimic the cochlea’s propagation of low-frequency signals over a broad region while also modeling the rapid damping of high-frequency signals. The cochlea employs a logarithmic frequency system, with varying regional resonances based on center frequencies and bandwidths. These frequency ranges form a cascaded filter chain, where each filter’s output is passed to the next. FAC manages high sound amplitudes within the cochlea. Sound entering the ear is amplified in the eardrum and middle ear. In the inner ear, high amplitudes are compressed, reducing the dynamic range. FAC responds rapidly to sudden signal changes, applying adaptive amplitude compression based on signal amplitude. Loud sounds are attenuated, while quiet sounds are amplified. This process, distinct from pre-emphasis, facilitates adaptation to diverse sound levels and protects ear structures from sustaining damage to due loud noises. After frequency decomposition and amplitude compression, the signal is optimized for neural coding and made suitable for audio processing applications. Biologically reliable and robust, CARFAC accurately models inner ear function. It is highly effective in applications requiring human ear emulation, preserving the naturalness of the signals by retaining both temporal and frequency information. It also has many uses in speech processing, hearing aids, cochlear implants, music and sound analysis, and acoustic modeling. While it is similar to Lyon and gammatone filters, CARFAC possesses several key differences. Gammatone and Lyon filters use fixed frequency bands, whereas CARFAC employs stepped asymmetric resonators. Gammatone requires external amplitude compression, while Lyon integrates it. CARFAC uses fast, volume-adaptive amplitude compression, and its rapid, adaptive temporal processing mechanism is more specialized than Lyon and gammatone. These features make CARFAC more effective for speech processing and speech emotion recognition [1,44].

**Figure 3:**
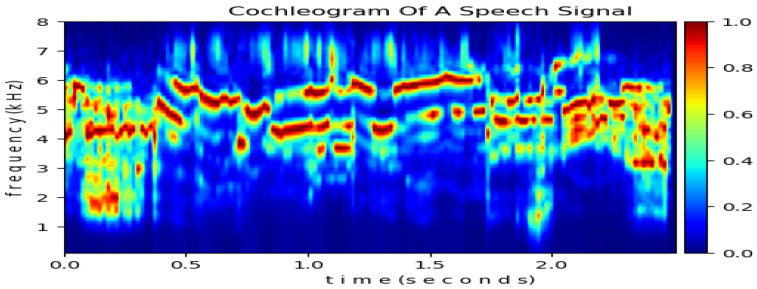
A sample cochleogram output from the ASED dataset.

Since 2011, the CARFAC model has been updated with new features. The output of CARFAC is called a cochleogram and is a time–frequency representation of signals such as the spectrogram depicted in Figure 3. In the CARFAC 2024 model, the AC coupler was moved to the exit of the basilar membrane from the inner hair cell to suppress the low frequencies and DC component. An open-loop bug was fixed to prevent ramping, and a linear CAR option was included by setting the nonlinear output of OHC to 1.0 when the AGC output is constant. Additionally, the previous two-capacitor IHC model was replaced with a new one, and a delay-per-stage option was added to ensure that the inputs of each stage depend solely on the outputs of the previous time steps. The gain factor between the cascaded stages was updated to stabilize the low-frequency end of the transfer function. The AGC spatial smoothing filter option was removed, and sensorineural hearing loss was modeled by amending the OHC_Health coefficient vector. One stage of the linearized CARFAC transfer function can be modeled with the Laplace Transform using the variable *s*. In this representation, the roots of the numerator are zero pairs, and the roots of the denominator are pole pairs, as governed by the following formula [36]:

(1) $$H\left(s\right)=\frac{\frac{s^{2}}{w_{z}^{2}}+\frac{2\xi_{z}s}{w_{z}}+1}{\frac{s^{2}}{w_{p}^{2}}+\frac{2\xi_{p}s}{w_{p}}+1}$$

where $w_{z}$ is the natural frequency of zeros, $w_{p}$ is the natural frequency of the poles, $\xi_{z}$ is the damping ratio of zeros, and $\xi_{p}$ is the damping ratio of the poles.

**Figure 4:**
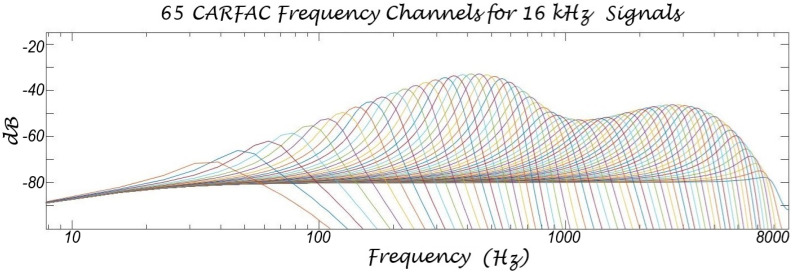
CARFAC 24 frequency bands used in the experiments. CARFAC 24 uses 65 bandpass filters for 16 kHz sampling rate. CARFAC 24 is significantly more efficient and natural than Mel filters.

**Figure 5:**
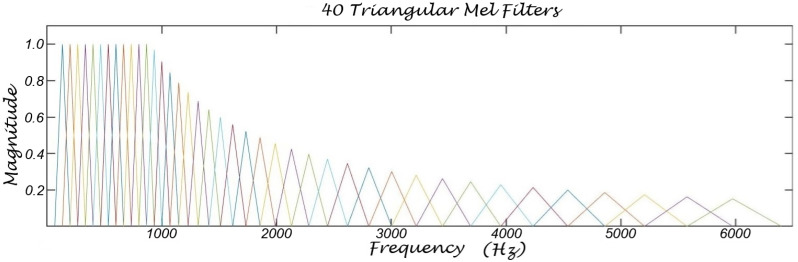
Mel filters illustration with 40 triangular bandpass filters.

Mel filters—a series of bandpass filters designed to model the frequency perception of the human hearing system—are frequently used in various applications, including speaker identification, speech recognition, speech keyword detection, speaker diarization, and speech emotion recognition. The human hearing system processes sounds by frequency and has variable sensitivity across different frequency regions. Sound processing occurs within the basilar membrane, a non-homogeneous structure that is stiff at the base and becomes progressively softer toward the apex. High-frequency sound components cause maximum oscillations at the base, while lower-frequency components are processed toward the tip. These frequency regions overlap, with each region creating a ripple effect in adjacent regions. Mel filters are compatible with this model of the basilar membrane and are effective in speech processing. However, it is generally acknowledged that they are not an exact model of human cochlear function, but a simplified version engineered for speech recognition compared to CARFAC 24 as shown in Figure 4 and Figure 5. Mel filters facilitate speech processing applications by extending the lower-frequency bands to avoid pitch-related features, which are often considered less informative in speech recognition applications [1]. However, in speech emotion processing and analysis, low-frequency components, particularly pitch and its temporal characteristics, are crucial for emotion classification and cannot be ignored. Mel filters are triangular-shaped bandpass filters with bandwidths typically spaced linearly up to 1000 Hz and logarithmically spaced thereafter.

MFCCs (Mel-Frequency Cepstral Coefficients) are another important feature extraction technique derived from Mel filters using the Discrete Cosine Transform (DCT) [45]. DCT extracts the most important components from a feature vector. During speech processing, MFCC typically uses 13 of the 40 DCT coefficients. Mel filter and MFCC calculations involve dividing the signal into overlapping chunks; applying the Fast Fourier Transform (FFT) to each chunk; calculating the energy under each Mel filter band; taking the logarithm to compress the dynamic range; applying the DCT; and, finally, selecting the first 13 coefficients for MFCC. Although MFCC uses fewer coefficients than Mel filters, it can achieve similar or even better performance in some speech processing applications. However, with the rise of deep learning, Mel filters often significantly outperform MFCCs in speech recognition tasks. However, when it comes to speech emotion recognition, MFCC achieves a state-of-the-art performance.

## 4. Experimental Design

### 4.1. Datasets

In this study, the newly introduced NEMO [46] and the ASED (Amharic Speech Emotion Dataset) [47] simulated emotional speech datasets are used to implement SER applications. The NEMO dataset contains 749 angry, 769 sad, 749 happy, 809 neutral, 669 surprise, and 736 fearful speech samples from Polish speakers. The 16-bit, mono-channel sound files are sampled at 24,000 Hz. The NEMO dataset includes nine native Polish speakers (five males and four females) aged 20–30. The audio samples have an average length of 2.47 s, a maximum of 5.65 s, a minimum of 0.86 s, and a standard deviation of 0.65 s, comprising a total duration of 3.07 h. Christop et al. [46] achieved 59.64% accuracy with SVM and 83.95% using Random Forest classifiers with 80% training data and 20% test data, using 20 MFCC features. No speaker-independent criteria were applied. Meanwhile, in our study, we use speaker-independent experiments and cross-validation, a common practice in emotional speech studies to prevent overfitting. The dataset comprises words and sentences commonly used in daily speech to provide a better phonetic representation of the Polish language. The NEMO dataset is well balanced in terms of the speakers’ gender, the number of samples per speaker, and the number of samples per emotional class.

Skowronski et al. [48] experimented on the CREMA-D, IEMOCAP, RAVDESS, SAVEE, TESS, and NEMO datasets using ResNet, Transformers, and various LSTM models with Mel spectrograms for classification. Their models used 80:10:10 training, validation, and test splits. They also performed multimodal emotion analysis using textual features. In speech emotion recognition experiments with the NEMO dataset, they achieved 84.60% accuracy using a parallel 2D CNN Transformer encoder model.

Our second dataset, the ASED, is also relatively new. It is the first dataset in Amharic, spoken in Ethiopia, and consists of emotional speech samples: 486 angry, 522 neutral, 470 sad, 510 fearful, and 486 happy. The 16,000 Hz sampled audio files have a total duration of 2.10 h, with a mean length of 3.06 s, a maximum of 3.9 s, a minimum of 2 s, and a standard deviation of 0.44 s. There are 65 speakers (20 females and 45 males) vocalizing 27 different sentences and various Amharic dialects. The speakers include 20 professional actors, 26 semi-professional actors, and 19 amateurs, aged 20–40. The speakers were twenty-one postgraduates, twenty-one university students, and twenty-three businesspeople. The dataset has a Fleiss Kappa value of 0.8, indicating high agreement among the eight referees. While the ASED is balanced in terms of the number of samples per emotion class, it is less evenly distributed in terms of speaker gender and the number of emotional class samples per gender. Retta et al. [47] used ResNet50, AlexNet, and VGG models with varying speakers for training and testing in speaker-independent implementations. They calculated Mel spectrograms with 128 channels and MFCC features with 40 coefficients. They used the ASED, EmoDB [49], RAVDESS [50], and URDU [51] speech emotion databases, achieving 84.76% accuracy in monolingual experiments with the VGG model on the ASED and 66.67% in cross-lingual experiments using the ASED for training and EmoDB for testing, also with the VGG [52] model.

### 4.2. Deep Learning Architectures Used in the Experiments

**Figure 6:**
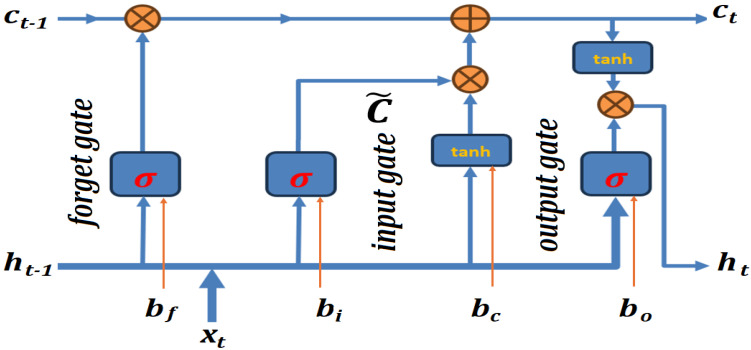
LSTM structure.

This study uses LSTM, VGG19, TDConvLSTM, and SVM-SMO classifiers. The artificial network community took a considerable amount of time to address the challenge of vanishing and exploding gradients, but eventually, LSTM was proposed by Hochreiter and Schmidhuber to resolve these issues [53]; its structure is depicted in Figure 6. When training LSTM, the value of the forget gate is critical, as the derivative of $c^{t}$ is taken with respect to $c^{t-1}$ during the backpropagation. The LSTM model is run as 2 LSTM layers with 512 and 256 hidden units, respectively. The LSTM layers are followed by 3 fully connected layers with 512, 256, 128 hidden units, respectively, and, finally, another fully connected softmax layer which outputs probability distribution of each class. Each dense unit is followed by a 0.25 dropout layer. The general formulae for the LSTM are as follows [54]:

(2) $${\tilde{c}}^{t}=g\left(W_{c}x^{t}+R_{c}h^{t-1}+b_{c}\right)\;block\;input\;i^{t}=\sigma \left(W_{i}x^{t}+R_{i}h^{t-1}+b_{i}\right)\;input\;gate\;f^{t}=\sigma \left(W_{f}x^{t}+R_{f}h^{t-1}+b_{f}\right)\;forget\;gate\;c^{t}=i^{t}⊙{\tilde{c}}^{t}+f^{t}⊙c^{t-1}\;memory\;{\;o}^{t}=\sigma \left(W_{o}x^{t}+R_{o}h^{t-1}+b_{o}\right)\;output\;gate\;h^{t}=o^{t}⊙g\left(c^{t}\right)\;block\;output$$

⊙: dot product, σ sigmoid activation, g hyperbolic tangent.

**Figure 7:**
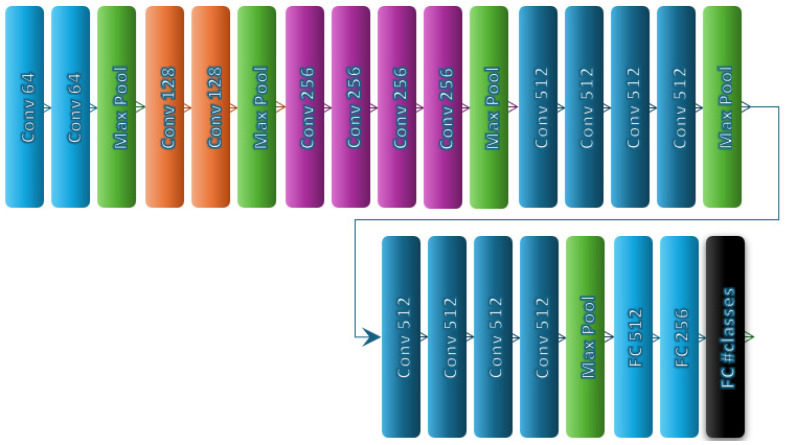
VGG19 model architecture.

After the advent of deep learning, CNNs (Convolutional Neural Networks) took the field by storm. VGG19, a pioneering and highly successful CNN model developed by the Visual Geometry Group, is a prime example. As the name suggests, it comprises 19 deep layers: 16 convolutional layers followed by 3 fully connected dense layers, as illustrated in Figure 7. Among the other powerful models used for sequence data with spatial dependencies in deep learning is Time Distributed Convolutional LSTM (TDConvLSTM), the structure of which is presented in Table 1.

**Figure 8:**
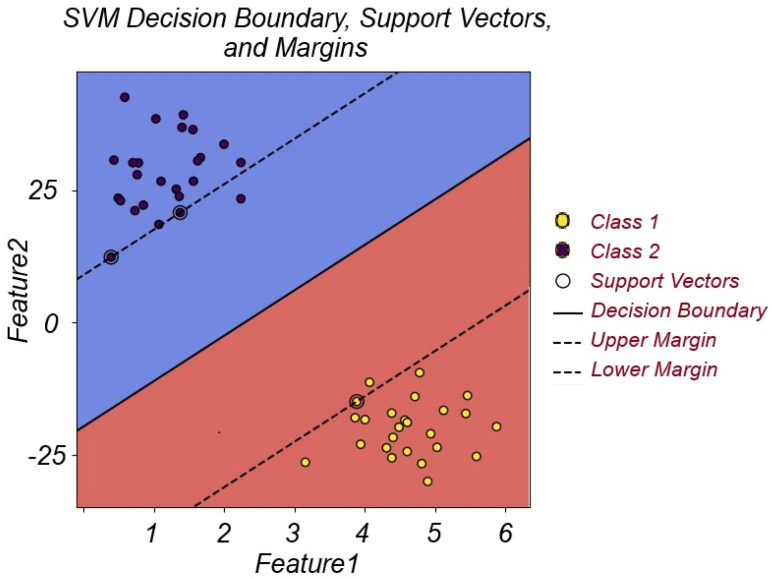
SVM’s support vectors, decision boundary, and margins.

**Figure 9:**
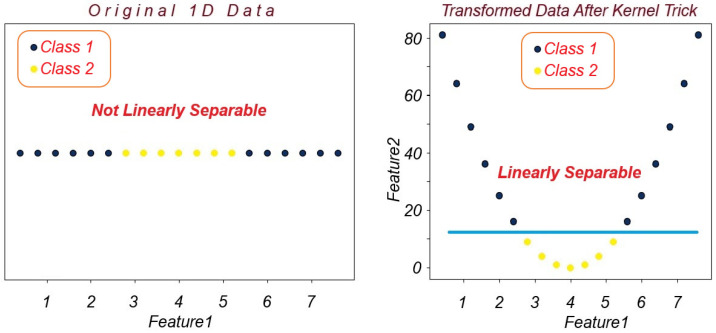
SVM uses kernel trick to make the data linearly separable.

Our final classifier is SVM [55], a powerful algorithm used for classification and regression. SVM attempts to obtain the best hyperplane in order to separate the different classes of data, as depicted in Figure 8. The data points closest to the hyperplane are called support vectors, and for optimal classification, the distance between the hyperplane and support vector data points should be maximized. SVM is primarily designed for linearly separable data; however, a technique called kernel trick enables SVM to classify nonlinear data. Kernel trick moves the data into a higher-dimensional hyperspace in which data points are linearly separable, as shown in Figure 9. Sequential Minimal Optimization [56] is an algorithm to efficiently tackle the quadratic programming problem of training SVMs. SMO uses a divide-and-conquer approach, splitting the problem into smaller problems which are easier to manage. SMO is particularly useful in large datasets. The most popular kernel functions for SVM training are Radial Basis Function and Polynomial kernels, which are used to handle nonlinear classification problems. The RBF kernel measures the distance between the data points and draws nonlinear decision boundaries in a high-dimensional space. The polynomial kernel, on the other hand, creates a high-dimensional space using polynomial combinations from the data features.

## 5. Results

In this section, we present the experimental results obtained for the ASED and the NEMO dataset using Support Vector Machines (SVMs), Time-Distributed Convolutional Neural Networks, VGG19, and LSTM networks. SVM classifications were implemented using Sequential Minimal Optimization (SMO) with Radial Basis Function (RBF) and default parameters within the Weka 3.8.6 toolkit [57]. For the Radial Basis Function kernel, the default parameter set is c=1,γ=0.01. In this study, the RBF kernel significantly outperformed the Polynomial kernel; however, the RBF kernel was too slow compared to the polynomial kernel. Given the moderate dataset size, high feature dimensionality, and small number of classes, the capabilities of SVMs were effectively demonstrated. Feature extraction was performed using the Auditory Modeling Toolbox [58] with the default parameter set for the CARFAC 24 system. CARFAC 24 generated 2905 features, including first-order delta coefficients and 22 statistical functions. Emotional speech utterances, sampled at 16,000 Hz, were divided into 31.25-millisecond chunks with a 50% overlap. Deep learning models were trained in Keras [59] using the Adam optimizer [60], with 100 epochs and a learning rate of 0.001. Experiments were conducted on 40 GB Colab A100 GPU with 83 GB RAM. Speaker-dependent implementation results are presented in Table 2 and Table 3, comparing SVM-SMO, TDConvLSTM, VGG19, and LSTM models with previous studies on the ASED and the NEMO dataset. For comparison with previous work [47,48], the datasets were split into 80% training and 20% testing sets, whereas for SVM implementations, we used 10-fold stratified cross-validations. CARFAC 24 outperformed [48] when used in tandem with the TDConvLSTM model.

**Figure 10:**
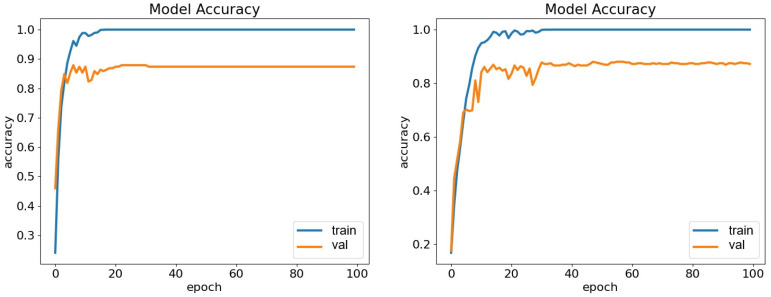
Accuracy plots of TDConvLSTM classifier for NEMO (left) and ASED (right) datasets in speaker-dependent experiments.

**Figure 11:**
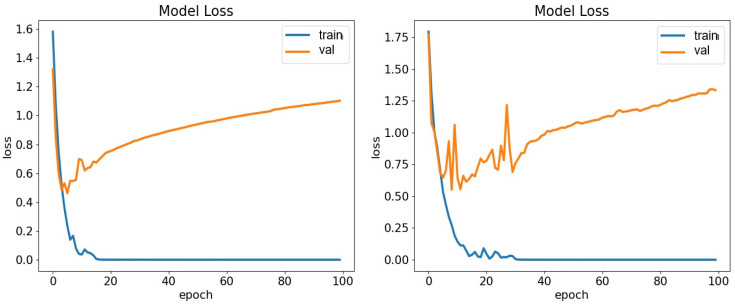
Loss plots of TDConvLSTM classifier for NEMO (left) and ASED (right) in speaker-dependent experiments.

**Figure 12:**
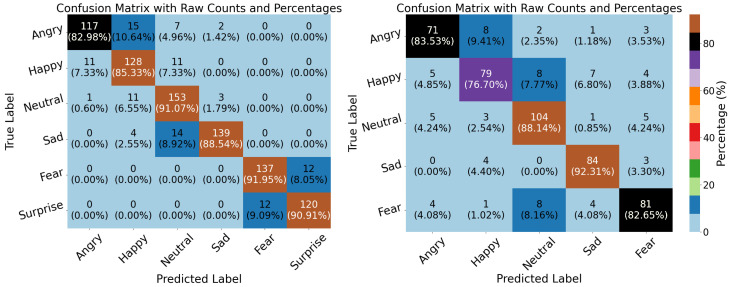
Confusion matrices of CARFAC 24 in NEMO dataset (left) and ASED (right) for the TDConvLSTM classifier in speaker-dependent experiments.

**Figure 13:**
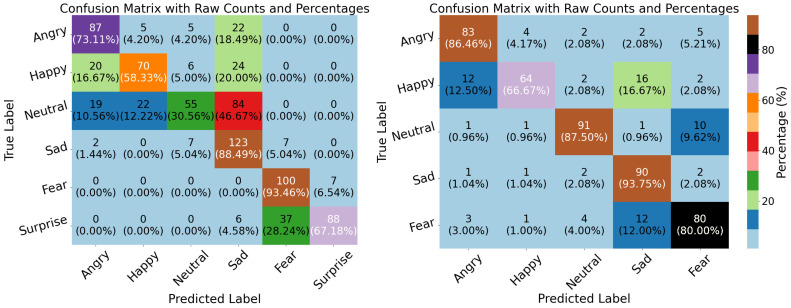
Speaker-independent confusion matrices of CARFAC 24 in NEMO dataset (left) and ASED (right) for TDConvLSTM classifier.

In our experiments, performance was evaluated based on accuracy (ACC), precision, recall, F1-score, and Kappa. Note that the Kappa metric is not provided by the Weka toolkit. The TDConvLSTM model outperformed the SVM-SMO, LSTM, and VGG19 models on both the ASED and the NEMO dataset. A key advantage of CARFAC 24 was its consistent performance across all classifiers and both datasets. When applied to the ASED, the TDConvLSTM model with CARFAC 24 features achieved 84.64% accuracy. Another strength of CARFAC 24 is the stability of its accuracy and loss during training and validation. It rapidly achieved optimal performance with minimal fluctuations for both datasets, as shown in Figure 10 and Figure 11. The confusion matrices for the TDConvLSTM model on the NEMO dataset and the ASED for speaker-dependent and speaker-independent experiments are presented in Figure 12 and Figure 13, respectively.

The speaker-independent results for the ASED and the NEMO dataset, using SVM-SMO, TDConvLSTM, VGG19, and LSTM models, are presented in Table 4 and Table 5, respectively. CARFAC 24 achieved 85.97% accuracy for the ASED, surpassing the results in [47] with the SVM-SMO classifier using an RBF kernel. It also achieved 65.70% accuracy when applied to the NEMO dataset with the TDConvLSTM classifier. When applied to the ASED, SVM-SMO demonstrated strong performance compared to the deep learning models VGG19 and LSTM. Although VGG19 achieved high accuracy in speaker-dependent implementations, its performance was significantly weaker in speaker-independent implementations.

Finally, the previous studies involving the ASED [47] and NEMO [48] are compared in Table 6. CARFAC 24 achieved a superior performance for both datasets, particularly the NEMO dataset, with a significant margin of approximately 4% accuracy.

## 6. Limitations of CARFAC 24 System

This paper showed the advantages of the novel CARFAC 24 system as a more natural and biologically inspired cochlear model. CARFAC 24 has its downsides, including its extremely long feature extraction stage compared to that of MFCC and Mel filters or other speech filter bank systems. This is because CARFAC 24 aims to imitate the human cochlea in its exact natural form without using any shortcuts or discarding any information. This is crucial for manufacturing better cochlear implants that provide users with a natural auditory experience, as well as making human–robot interaction more organic. Moreover, computers are becoming increasingly powerful and less expensive by the day. Therefore, these disadvantages may have very little impact on the development of CARFAC 24-based applications. Another disadvantage is, of course, the number and shape of the model’s filters. CARFAC 24 uses Gaussian filters as opposed to triangular or trapezoidal filters, creating structural and computational challenges. Some modifications can be made to reduce the number of filters required by CARFAC 24 and speed up the feature extraction stage, which will be an interesting avenue to pursue in future research. It should also be noted at this point that the success of an SER system is not solely dependent on the number of extracted features. If we can obtain a rapid and compact learning model using a large number of features, this would be a better system. Moreover, there are some studies which have used Mel spectrograms, scalograms, and Mel filters directly without implementing statistical functions [61,62,63]. In these cases, the number of features can easily surpass that of the CARFAC 24 system.

The highlights of this paper can be summarized as follows:

- CARFAC 24 outperforms traditional methods.
- Time-Distributed Convolutional LSTM models achieve high accuracy.
- SVM-SMO is quite successful when used in tandem with RBF kernel.

## 7. Discussions

In this paper, a cochleogram-based implementation of SER has been achieved with different classifiers on two emotional speech datasets using the recently proposed CARFAC 24 model. The experiments can mainly be categorized into two sections. The first section involves the speaker-dependent part, where the data are split at random. On the six-class NEMO dataset, the classifiers—namely, SVM-SMO, TDConvLSTM, VGG19, and LSTM—have similar performances, with LSTM lagging slightly behind. TDConvLSTM demonstrated the best performances in terms of accuracy when applied to both NEMO and the ASED. SVM-SMO achieved a performance highly similar to that of TDConvLSTM in the ASED; however, when applied to the NEMO dataset, TDConvLSTM and VGG19 significantly outperformed the SVM-SMO and LSTM classifiers. VGG19 also achieved the best precision when applied to the ASED. Meanwhile, VGG19 and TDConvLSTM proved powerful when utilized as part of the speaker-dependent test for the NEMO dataset. They obtained near-identical results, significantly outpacing SVM-SMO and LSTM in terms of accuracy. Classification algorithms are usually quite successful and highly accurate in speaker-dependent experiments. This highlights a serious problem known as overfitting. All classifiers are prone to overfitting, and a solution to this issue should be sought as part of the experimental design. One of the best techniques in speech processing applications is preventing the classifiers from memorizing speaker-dependent features. This can be achieved by hiding the test data from the classifiers during the training period; however, it often has a significant negative impact on the performances of the classifiers. Some striking evaluations arise when the results of speaker-independent experiments are analyzed. First of all, SVM-SMO achieves a very strong performance, particularly when applied to the ASED, surpassing all others by a very significant margin and exceeding the speaker-dependent implementation accuracies on the ASED. In the speaker-independent experience, the performance of VGG19 is quite disappointing with regard to both the ASED and the NEMO dataset. Another interesting observation is the significant performance loss in the NEMO dataset during speaker-independent experiments. When applied to the NEMO dataset, TDConvLSTM achieved the best score; however, it still exhibited more than a 20% performance degradation compared to the speaker-dependent case. Another key point is that all metrics were fairly stable and close to one another for a specific classifier both in speaker-dependent and independent tests. When the confusion matrices of speaker-dependent experiments were investigated, as shown in Figure 12, we observed that for the NEMO dataset, the class-wise accuracies differed by around 10%, with the Fear class achieving the greatest accuracy (91.95%). The surprise and fear classes were not mixed with other classes. In the speaker-dependent experiments involving the ASED, the happy class had the worst detection rate, with 76.70% accuracy, as shown in Figure 12. The best detection results were achieved for the Sad class in the ASED, and the class-wise accuracies for this dataset varied quite broadly, with 92.31% for the sad class and 76.70% for the happy class—a discrepancy of more than 15%—with the happy class once again given the worst detection rate. In the confusion matrices of speaker-independent experiments involving the NEMO dataset, fear was the best detected emotion, with 93.46% accuracy, whereas the neutral class was the worst detected class, with 30.56% accuracy as graphed in Figure 13. Fear and surprise were rarely confused with other classes. In speaker-independent tests involving the ASED, the sad class once again achieved the best detection rate, with 93.75% class-wise accuracy, whereas happiness had the poorest detection rate, with 66.67% accuracy. The gap between class-wise accuracies was lower for the ASED compared to the NEMO dataset. Finally, if we evaluate Table 6 in relation to the NEMO dataset, CARFAC 24 demonstrated exceptional performance compared to that achieved in previous studies.

## 8. Conclusions

This study introduces a novel approach by applying the CARFAC 24 model and its cochleogram output to speech emotion recognition and comparing the results with previous work. CARFAC 24 is designed to perfectly mimic the structure of the human cochlea, in contrast with the Mel scale, which has never been considered an accurate representation of the ear. While Mel filters and MFCCs were useful in the early days of machine learning due to computational limitations, today’s powerful machines no longer require such simplifications, which can compromise model performance. It is time to replace these older models with newer, more effective versions. Our experiments demonstrate that CARFAC 24 significantly outperforms previous studies across all experiments. We evaluated SVM-SMO, TDConvLSTM, VGG19, and LSTM models, and determined that SVM-SMO and TDConvLSTM achieved superior performances in SER applications. Despite being an older classifier, when conditions are favorable, SVM-SMO can be highly effective. The experimental results clearly indicate CARFAC 24’s utility in emotional speech studies; it easily surpasses other implementations using Mel filters or MFCCs. In this study, CARFAC 24 uses 65 bands with a 16 kHz sampling rate, and the calculation of each band is dependent on the previous band’s calculation. While we used the standard 65 filters, CARFAC 24’s parameters can be adjusted to create a different number of filters (e.g., 48 or 36), which may achieve similar or even better performance in SER applications. In future, researchers could explore the use of CARFAC 24 for other speech processing applications and incorporate feature selection algorithms into the model. Utilizing CARFAC 24 for speech emotion analysis will improve the efficacy of human–computer interaction, making it more natural.

## Figures and Tables

**Table 1:** Time-Distributed Convolutional LSTM architecture.

Layer	Output Shape	#Parameters
Input	(None, 1, 44, 66, 1)	0
Time Distributed Conv2D (256, (3 × 3), “relu”)	(None, 1, 44, 66, 256)	2560
Time Distributed Conv2D (256, (3 × 3), “relu”)	(None, 1, 44, 66, 256)	590,080
Time Distributed MaxPool (2, 2)	(None, 1, 22, 33, 256)	0
Time Distributed (Flatten)	(None, 1, 185,856)	0
LSTM (512)	(None, 1, 512)	381,683,712
LSTM (256)	(None, 1, 256)	787,456
LSTM (128)	(None, 128)	197,120
Drop Out (0.25)	(None, 128)	0
Flatten	(None, 128)	0
Dense (512)	(None, 512)	66,048
Drop Out (0.5)	(None, 512)	0
Dense (#classes)	(None, 6)	3078

**Table 2:** Speaker-dependent experiments on ASED dataset.

	ACC	Precision	Recall	F1	Kappa
**SVM-SMO**	84.51	84.60	84.51	84.51	**-**
**TDConvLSTM**	**84.64**	84.59	**84.64**	**84.56**	**80.74**
**VGG19**	84.24	**84.87**	84.24	84.23	80.25
**LSTM**	83.23	83.37	83.23	83.18	78.99

**Table 3:** Speaker-dependent experiments on NEMO dataset.

	ACC	Precision	Recall	F1	Kappa
**SVM-SMO**	86.72	86.90	86.72	86.80	**-**
**TDConvLSTM**	**88.51**	**88.83**	**88.51**	**88.57**	**86.19**
**VGG19**	88.40	88.46	88.40	88.35	86.07
**LSTM**	82.60	84.33	82.60	82.55	79.14

**Table 4:** Speaker-independent results of experiments on the ASED dataset.

	ACC	Precision	Recall	F1-Score	Kappa
**SVM-SMO**	**85.97**	**86.70**	**86.00**	**85.90**	**-**
**TDConvLSTM**	82.93	83.77	82.93	82.77	78.66
**VGG19**	79.67	81.60	79.67	79.39	74.58
**LSTM**	82.11	84.10	82.11	82.06	77.64

**Table 5:** Speaker-independent results of experiments on the NEMO dataset.

	ACC	Precision	Recall	F1-Score	Kappa
**SVM-SMO**	64.07	67.40	64.07	63.60	-
**TDConvLSTM**	**65.70**	**70.95**	**65.70**	**64.41**	**58.98**
**VGG19**	60.67	64.18	60.67	60.26	52.92
**LSTM**	64.45	65.11	64.45	63.86	57.33

**Table 6:** Comparison with previous studies on ASED and NEMO datasets.

	ASED Dataset
**CARFAC 24 (SVM-SMO RBF Kernel)**	**85.97% (ACC)**
Retta et al. [47]	84.72% **(ACC)**
	**NEMO Dataset**
**CARFAC 24 (TDConvLSTM)**	**88.51% (ACC)**
Skowronski et al. [48]	84.60% **(ACC)**

## Data Availability

Data are available at https://github.com/cevparlak/CARFAC-24 (accessed on 16 February 2025). The ASED is available at https://github.com/Ethio2021/ASED_V1 (accessed on 16 February 2025). The NEMO dataset is available at https://github.com/amu-cai/nEMO (accessed on 16 February 2025).

## Abbreviations

The following abbreviations are used in this manuscript:

ADAM	Adaptive Moment
AGC	Automatic Gain Control
ASED	Amharic Speech Emotion Dataset
CARFAC	Cascade of Asymmetric Resonators with Fast-Acting Compression
DCT	Discrete Cosine Transform
FFT	Fast Fourier Transform
FPGA	Field Programmable Gate Array
HWR	Half Wave Rectifier
IHC	Inner Hair Cell
LSTM	Long Short Term Memory
MFCC	Mel Frequency Cepstral Coefficients
OHC	Outer Hair Cell
PZFC	Pole-Zero Filter Cascade
RBF	Radial Basis Functions
ResNet	Residual Network
SER	Speech Emotion Recognition
SM	Scala Media
ST	Scala Tympani
SV	Scala Vestibuli
SVM	Support Vector Machines
SMO	Sequential Minimal Optimization
TDConvLSTM	Time Distributed Convolutional LSTM
Weka	Waikato Environment for Knowledge Analysis
VGG	Visual Geometry Group
